# Lorca's hospital evacuation and medical attention by the emergency department

**DOI:** 10.1186/cc12215

**Published:** 2013-03-19

**Authors:** L Escobar Alvaro, J Jimenez Gonzalez, A Pelaez Ballesta, A Corbatón Anchuelo

**Affiliations:** 1 Hospital Rafael Mendez, Lorca, Spain; 2 Hospital Clínico San Carlos, Madrid, Spain

## Introduction

On 11 May 2011 two moderate magnitude earthquakes hit the city of Lorca, southeast Spain. They caused 11 deaths, including two pregnant women and their babies, more than 350 injured, and moderate or severe damage to 80% of the building in the city, including Lorca's public hospital that had to be evacuated.

## Methods

A descriptive study of the files of Lorca's hospital and the clinical records of patients that attended our service in the 20 hours following the second earthquake on 11 May.

## Results

**Figure 1:**
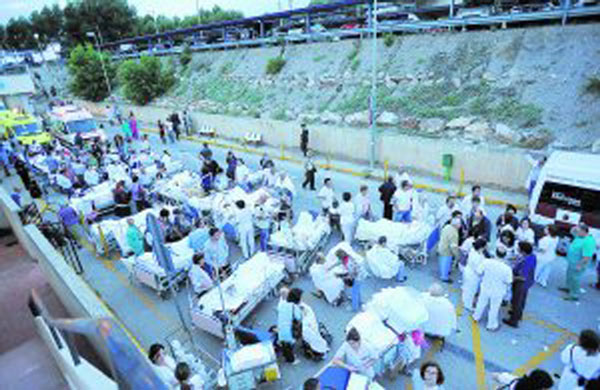
**Hospital evacuation**.

**Figure 2:**
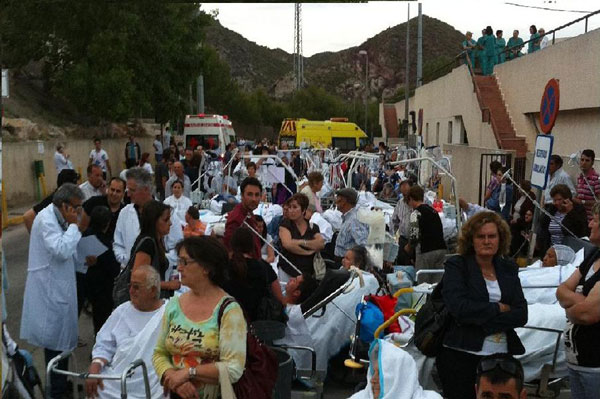
**Emergency service taking care of patients during evacuation**.

A total of 225 patients were relocated. Simultaneously 224 patients were treated by the emergency service, until the evacuation was completed and after then, from 10:00 pm to 3:00 pm the next day, 47 more in a field hospital placed just outside the hospital building. See Figures 1 and 2.

## Conclusion

In less than 3 hours 225 patients were evacuated and 224 were given attention by the emergency service of Lorca's hospital, with the support of personnel from other services of the hospital. The emergency service of the hospital continues to be operative in the building until evacuation is completed and in a field hospital later.

